# Plant-Based Medically Tailored Groceries From a Food Pantry and Adult Cardiometabolic Outcomes

**DOI:** 10.5888/pcd23.260023

**Published:** 2026-10-01

**Authors:** Jacob B. Mirsky, Allison J. Wu, Mandy Luo, Meghan E. Perkins, Joseph J. Locascio, Marie-France Hivert, Rachel Burgun, Michele Fronk Schuckel, Elsie M. Taveras, Lauren Fiechtner

**Affiliations:** 1 Division of General Internal Medicine, Massachusetts General Hospital, Boston, Massachusetts; 2 Department of Medicine, Harvard Medical School, Boston, Massachusetts; 3 Boston Children’s Hospital, Boston, Massachusetts; 4 Department of Pediatrics, Harvard Medical School, Boston, Massachusetts; 5 Division of General Academic Pediatrics, Mass General Brigham for Children, Boston, Massachusetts; 6 Department of Neurology, Harvard Medical School, Boston, Massachusetts; 7 Department of Population Medicine, Harvard Pilgrim Health Care Institute, Boston, Massachusetts; 8 Diabetes Unit, Massachusetts General Hospital, Boston, Massachusetts; 9 The Greater Boston Food Bank, Boston, Massachusetts

## Abstract

This study examined the association of weekly plant-based medically tailored groceries (MTGs) with cardiovascular-kidney-metabolic outcomes among patients with food insecurity. The intervention began on December 20, 2019, and ended on April 1, 2025 (275 weeks). Linear mixed-effects models estimated the dose-dependent association of MTGs received and the rates of changes in blood pressure, glycated hemoglobin A_1c_, weight, and body mass index from baseline to follow-up periods. Among 68 adults, during an average of 2.7 years, each 100 MTGs received was associated with a greater improvement from baseline to follow-up periods in the rates of changes in diastolic blood pressure (−3.21 mm Hg; 95% CI, −4.89 to −1.52), weight (−2.63 kg; 95% CI, −3.73 to −1.53), and body mass index (−1.04 kg/m^2^; 95% CI, −1.50 to −0.58).

**Summary**

**What is already known on this topic?**

Food Is Medicine (FIM) interventions such as medically tailored groceries (MTGs), medically tailored meals, and produce prescriptions have been shown to improve dietary quality and reduce food insecurity. Effects of FIM interventions on cardiovascular-kidney-metabolic (CKM) health outcomes have been mixed.

**What is added by this report?**

Most FIM intervention studies to date have focused on a duration of weeks or months, and few FIM interventions optimized nutritional quality. This report is unique in estimating the effect of plant-based MTGs during nearly 3 years of follow-up.

**What are the implications for public health practice?**

Plant-based FIM interventions are promising; to improve CKM health, future interventions should be of longer duration.

## Objective

Food insecurity is associated with lower dietary quality and cardiovascular-kidney-metabolic (CKM) health, including higher rates of hypertension, obesity, and type 2 diabetes (1). Food Is Medicine (FIM) interventions such as medically tailored groceries (MTGs), medically tailored meals, and produce prescriptions have proliferated in recent years to address food insecurity, with the goal of preventing and treating chronic diseases, and they have been shown to improve dietary quality and reduce food insecurity (2). However, the effect of FIM programming on CKM health outcomes is mixed (3).

Exploration of the dose effect will be as critical for FIM studies as they are for pharmaceutical interventions. Four key FIM dose factors that have likely limited the effect of FIM interventions are frequency, quality, quantity, and duration (2). FIM interventions could be strengthened by more frequent provisions of the most nutritious foods with greater quantity and a longer duration. For example, we previously observed that provision of weekly plant-based food packages was associated with decreased child body mass index (BMI) in a dose-dependent fashion over 19 months (2,4).

The objective of this study was to examine the dose-dependent association of frequent, nutritious, high-quantity plant-based packages of MTGs from a food pantry with long-term CKM outcomes in adults with food insecurity.

## Methods

The Massachusetts General Hospital (MGH) Revere Food Pantry is in a community-based health care center and provides weekly plant-based MTGs (2,4). Patients receiving MTGs are predominantly non-English-speaking people who have low socioeconomic status and use public health insurance (4,5). A dietitian initially guided food selection based on the US Department of Agriculture’s 2020 Dietary Guidelines, providing each family member 3 meals per day for a week. MTGs at the MGH Revere Food Pantry contain fresh fruits and vegetables, canned beans and/or nut butter, and whole grains.

This retrospective study included nonpregnant adults (aged ≥18 y) who 1) received at least 26 MTGs from December 20, 2019, through April 1, 2025; 2) had at least 1 baseline CKM measurement (up to 12 months before receipt of first MTG [ie, preintervention]; median no. of measurements = 4); and 3) had at least 1 follow-up CKM measurement (from 6 months after receipt of first MTG to 3 months after receipt of last MTG [ie, postintervention]; median no. of measurements = 8) for at least 1 prespecified outcome.

Primary outcomes included systolic and diastolic blood pressure and glycated hemoglobin A_1c_ (HbA_1c_). Secondary outcomes included BMI and weight. Sensitivity analyses evaluated outcomes among adults with an abnormal baseline value. The study was approved by the Mass General Brigham Institutional Review Board as exempt research.

### Statistical methods

We extracted longitudinal outcome measures from the electronic health record. Height and weight data were cleaned using the anthropometric data cleaning algorithm developed by Daymont et al (6). Linear mixed-effects models estimated the association between participant-level MTGs and differences in the rates of change in outcomes from baseline to follow-up periods. A participant-specific random intercept accounted for within-individual correlation among repeated outcome measurements. Unadjusted Model 1 included fixed effects for MTGs received (measured in 100s of packages), study period (baseline period vs follow-up period), and their interaction. Model 2 additionally adjusted for age at baseline, sex, baseline outcome values, and weeks between baseline and each follow-up outcome measurement. Model 3 additionally adjusted for COVID-19 pandemic periods (ie, before, during, and after) (7,8).

## Results

Among 68 adult participants (Figure), 47 (69.1%) were female and mean (SD) age was 57.4 (13.7) years. Twenty-two (37.3%) participants identified as Hispanic/Latino. Fifty (80.6%) had a baseline BMI of 25 or more; mean (SD) systolic blood pressure was 130.3 (20.0) mm Hg (hypertension stage 1 range); mean (SD) diastolic blood pressure was 76.5 (8.4) mm Hg; and mean (SD) HbA_1c_ was 6.9% (1.8%) (diabetes range). Most participants had public health insurance (72.1%). The mean (SD) number of MTGs received during the observation period was 97.6 (66.2) over an average of 142 weeks, indicating that participants on average received MTGs approximately every 1.4 weeks for 2.7 years (Table 1).

**Figure:**
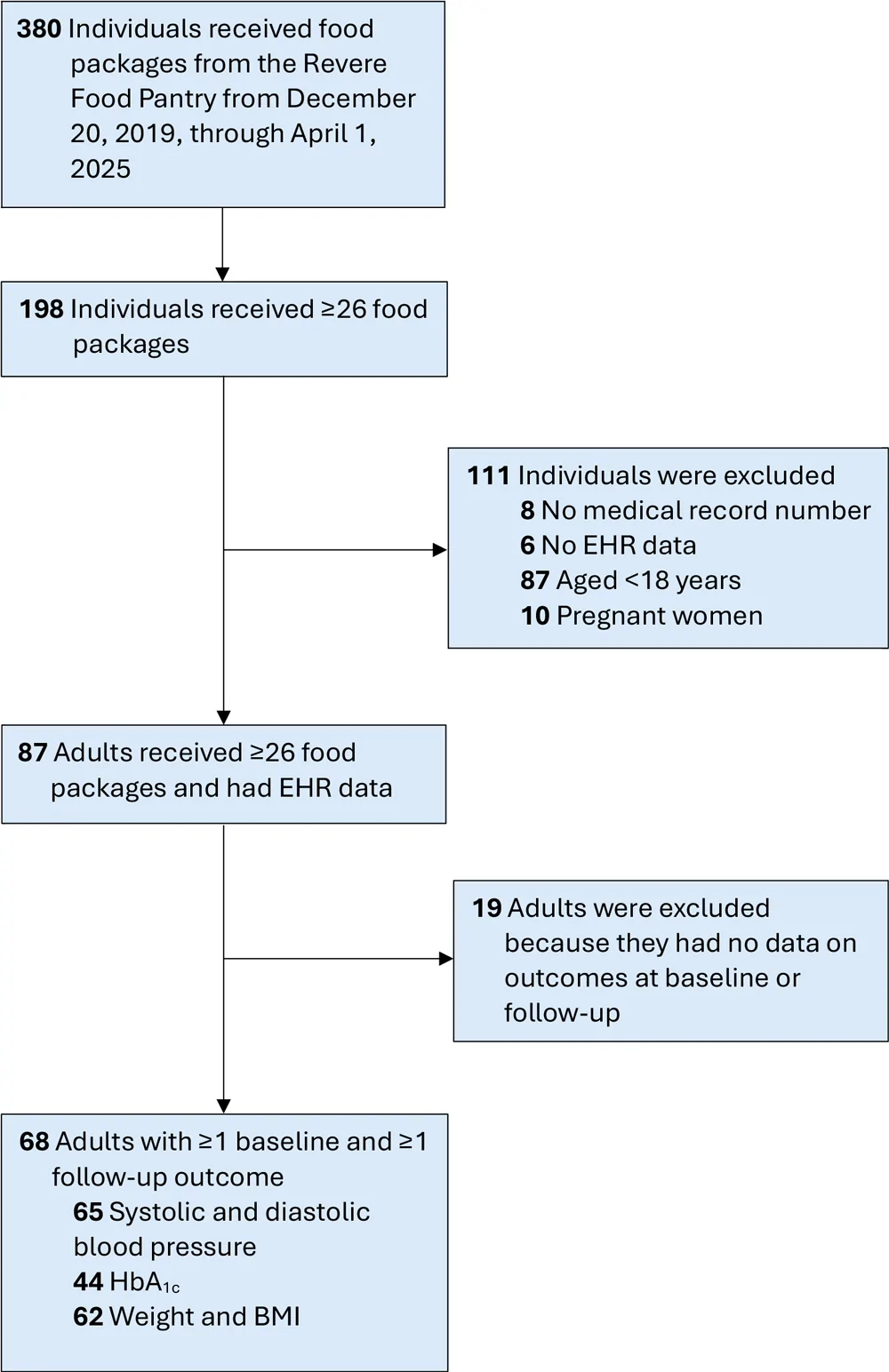
Participants in a plant-based Food Is Medicine intervention at the Massachusetts General Hospital Revere Food Pantry, December 2019–April 2025. Abbreviation: BMI, body mass index; EHR, electronic health record; HbA1c, glycated hemoglobin A_1c_.

**Table 1:** Baseline^a^ Characteristics of Participants (N = 68) in a Plant-Based Food Is Medicine Intervention at a Community-Based Health Care Center, Revere, Massachusetts, 2019–2025

Characteristic	No. in sample	Value
**Demographic**		
**Age at time of first food package, y**		
Mean (SD)	68	57.4 (13.7)
Median (IQR)		56.2 (48.9–67.8)
**Sex, no. (%)**		
Female	68	47 (69.1)
Male		21 (30.9)
**Race and ethnicity, no. (%)**		
Hispanic or Latino	59	22 (37.3)
Non-Hispanic Black		8 (13.6)
Non-Hispanic White		25 (42.4)
Non-Hispanic Asian		4 (6.8)
**Type of health insurance, no. (%)**		
Public insurance (eg, Medicaid, Medicare)	68	49 (72.1)
Private insurance		19 (27.9)
**Food packages**		
**No. of weeks food packages received from December 20, 2019, through April 1, 2025**		
Mean (SD)	68	97.6 (66.2)
Median (IQR)		71.0 (43.0–141.5)
**No. of weeks between first and last food package date December 20, 2019, through April 1, 2025**		
Mean (SD)	68	142.5 (75.8)
Median (IQR)		141.0 (75.0–197.0)
**No. of weeks of food packages received in the first 6 months of intervention period**		
Mean (SD)	68	20.7 (5.0)
Median (IQR)		22.0 (18.0–24.0)
**Health outcomes**		
**Systolic blood pressure**		
Mean (SD), mm Hg	65	130.3 (20.0)
Median (IQR), mm Hg		126.0 (118.0–138.0)
≥130 mm Hg, no. (%)		31 (47.7)
**Diastolic blood pressure**		
Mean (SD), mm Hg	65	76.5 (8.4)
Median (IQR), mm Hg		77.0 (72.0–80.0)
≥80 mm Hg, no. (%)		24 (36.9)
**Hemoglobin A_1c_**		
Mean (SD), %	44	6.9 (1.8)
Median (IQR), %		6.1 (5.6–8.7)
≥7%, no. (%)		16 (36.4)
**Weight, kg**		
Mean (SD)	62	83.6 (23.2)
Median (IQR)		80.7 (68.0–93.9)
**Body mass index, kg/m^2^**		
Mean (SD)	62	31.7 (7.9)
Median (IQR)		30.2 (27.0–35.7)
**Body mass index category**, **no. (%)**		
Underweight or normal weight (<25 kg/m^2^)	62	12 (19.4)
Overweight (25 to <30 kg/m^2^)		17 (27.4)
Obese (≥30 kg/m^2^)		33 (53.2)

a Baseline defined as up to 12 months before receipt of first food package.

For each 100 MTGs received, the rate of change in diastolic blood pressure was reduced by 3.21 mm Hg (95% CI, −4.89 to −1.52) from baseline to follow-up periods, adjusted for age, sex, baseline diastolic blood pressure, weeks of follow-up, and the COVID-19 pandemic (Table 2). We observed no analogous significant changes in systolic blood pressure or HbA_1c_ levels.

**Table 2:** Mixed Linear Models of Cardiovascular-Kidney-Metabolic Health Outcomes in a Plant-Based Food Is Medicine Intervention at a Community-Based Health Care Center, Revere, Massachusetts, 2019–2025^a^

Outcome	No. in sample	Model 1 (unadjusted)^b^		Model 2 (adjusted)^c^		Model 3 (fully adjusted)^d^	
		β interaction (95% CI)	*P* value	β interaction (95% CI)	*P* value	β interaction (95% CI)	*P* value
**Primary**							
Systolic blood pressure, mm Hg	65	0.22 (−2.04 to 2.49)	.85	0.56 (−1.80 to 2.90)	.64	−2.71 (−5.73 to 0.30)	.08
Baseline systolic blood pressure ≥130 mm Hg	31	−0.46 (−3.44 to 2.50)	.76	−0.18 (−3.30 to 2.85)	.91	−5.83 (−10.09 to −1.57)	.01
Diastolic blood pressure, mm Hg	65	−1.53 (−2.79 to −0.26)	.02	−1.48 (−2.79 to −0.16)	.03	−3.21 (−4.89 to −1.52)	<.001
Baseline diastolic blood pressure ≥80 mm Hg	24	−1.46 (−3.32 to 0.45)	.13	−1.68 (−3.61 to 0.23)	.09	−6.23 (−9.25 to −3.32)	<.001
**Secondary**							
HbA_1c_, %	44	−0.10 (−0.43 to 0.22)	.53	−0.05 (−0.37 to 0.27)	.78	−0.03 (−0.44 to 0.38)	.90
Baseline HbA_1c_ ≥7%	16	0.11 (−0.41 to 0.63)	.68	0.21 (−0.32 to 0.73)	.44	0.16 (−0.48 to 0.79)	.62
BMI, kg/m^2^	62	−1.10 (−1.42 to −0.77)	<.001	−1.11 (−1.45 to −0.76)	<.001	−1.04 (−1.50 to −0.58)	<.001
Baseline BMI ≥25 kg/m^2^	50	−1.13 (−1.51 to −0.75)	<.001	−1.17 (−1.56 to −0.76)	.001	−0.89 (−1.50 to −0.29)	.004
Weight, kg	62	−2.68 (−3.47 to −1.90)	<.001	−2.63 (−3.46 to −1.79)	<.001	−2.63 (−3.73 to −1.53)	<.001

Abbreviation: BMI, body mass index; HbA_1c_, glycated hemoglobin A_1c_; MTG, medically tailored groceries.

a Adult anthropometric data were cleaned using the anthropometric data cleaning algorithm developed by Daymount et al (6).

b Model 1 (unadjusted) included fixed effects for MTGs received (measured in 100s of packages), study period (baseline period vs follow-up period), and an interaction term between MTGs and study period. The results report the effect sizes of the interaction term (β = partial unstandardized regression coefficient). Baseline period was defined as up to 12 months before receipt of first MTG; follow-up period was defined as from 6 months after receipt of first MTG to 3 months after receipt of last MTG.

c Model 2 (adjusted) additionally adjusted for age (in years) at baseline, sex, baseline outcome value, and time (in weeks) between baseline and each outcome measurement.

d Model 3 (fully adjusted) additionally adjusted for COVID-19 pandemic based on World Health Organization definitions (pre-COVID-19: January 1, 2019–March 20, 2020, COVID-19: March 11, 2020–May 4, 2023, post-COVID-19: May 5, 2023–June 30, 2025) for each measurement time point.

In sensitivity analyses among those with high baseline systolic blood pressure (≥130 mm Hg), for each 100 MTGs received, the corresponding rate of change in systolic blood pressure was reduced by 5.83 mm Hg (95% CI, −10.09 to −1.57) from baseline to follow-up periods in fully adjusted models; among those with high baseline diastolic blood pressure (≥80 mm Hg), for each 100 MTGs received, the rate of change in diastolic blood pressure was reduced by 6.23 mm Hg (95% CI, −9.25 to −3.32).

For each 100 MTGs received, the rate of change in BMI from baseline to follow-up periods was reduced by 1.04 kg/m^2^ (95% CI, −1.50 to −0.58) and the rate of change in weight decreased by 2.63 kg (95% CI, −3.73 to −1.53), adjusted for age, sex, baseline BMI and weight (respectively), weeks of follow-up, and the COVID-19 pandemic. In sensitivity analyses among those with an elevated baseline BMI (≥25), for each 100 MTGs received, the rate of change in BMI was reduced by 0.89 (95% CI, −1.50 to −0.29) in fully adjusted models.

## Discussion

In this retrospective study of adults with food insecurity and poor CKM health receiving frequent, high-quality, high-quantity plant-based MTGs over an average of almost 3 years, we observed improvements in several markers of CKM health. In a dose-dependent fashion, the rates of changes in diastolic blood pressure, weight, and BMI were reduced; among those with high baseline values, the rates of changes in systolic blood pressure, diastolic blood pressure, and BMI were reduced. The observed reductions in blood pressure are especially encouraging given that hypertension is the leading risk factor for heart attacks, heart failure, and stroke (2). Most patients in this study had public health insurance, indicating that governmental programs may benefit from emphasizing potent FIM interventions to improve health outcomes and lower the total cost of care.

We found no estimated differences in the rates of changes in systolic blood pressure or HbA_1c_ among any patients, which is consistent with several other research studies on FIM interventions. A recent systematic review highlighted multiple randomized controlled trials of MTGs that similarly showed no significant differences in blood pressure or HbA_1c_ (2). Many factors that influence blood pressure and blood glucose control beyond food (eg, medication adherence, physical activity, psychological stress) likely mitigate the effect of FIM interventions. Additionally, participation in the MGH Revere Food Pantry might be associated with improved medication adherence, which could also affect CKM outcomes independent of the dose of MTGs.

Limitations of this study include lack of a control group and reliance on office-based biometrics, which can be inaccurate and inconsistent across multiple sites. Future studies would benefit from a randomized controlled design and precise measurements of frequent biometrics and potential confounders that could drive both increased use of MTGs and improved CKM outcomes, such as the severity of food insecurity, housing insecurity, and employment status. Another possible limitation of the study is that, although food packages are considered MTGs, a dietitian did not tailor recommendations for plant-based food items for each family or chronic medical condition. The findings from this study may not generalize to patient populations who are not connected to a community-based health care center such as MGH Revere, which offers many resources to support community health, such as community health workers.

Future FIM interventions should focus on longer-term plant-based food packages to improve CKM health. This study is notable for the changes observed over almost 3 years, indicating that FIM interventions may need to extend beyond weeks or months to see sustained, clinically meaningful results.
